# *STIL* Endows Oncogenic and Stem-Like Attributes to Colorectal Cancer Plausibly by Shh and Wnt Signaling

**DOI:** 10.3389/fonc.2021.581671

**Published:** 2021-08-12

**Authors:** Tapas Pradhan, Vikas Kumar, Evangeline Surya H, R. Krishna, Samu John, V. T. Jissa, S. Anjana, K. Chandramohan, S. Asha Nair

**Affiliations:** ^1^Cancer Research Program 4, Rajiv Gandhi Centre for Biotechnology, Trivandrum, India; ^2^Cardiovascular Diseases & Diabetes Biology, Rajiv Gandhi Centre for Biotechnology, Trivandrum, India; ^3^Achutha Menon Centre for Health Science Studies (AMCHSS), Sree Chitra Tirunal Institute for Medical Sciences and Technology, Trivandrum, India; ^4^Department of Surgical Oncology, Regional Cancer Centre, Trivandrum, India

**Keywords:** colorectal cancer, STIL, hedgehog signaling, cancer stem cell, drug resistance, β-catenin, prognosis STIL Oncogene in CRC

## Abstract

The discovery of a potent gene regulating tumorigenesis and drug resistance is of high clinical importance. *STIL* is an oncogene; however, its molecular associations and role in colorectal oncogenesis are unknown. In this study, we have explored the role of *STIL* gene in tumorigenesis and studied its molecular targets in colorectal cancer (CRC). *STIL* silencing reduced proliferation and tumor growth in CRC. Further, STIL was found to regulate stemness markers *CD133* and *CD44* and drug resistant markers *thymidylate synthase*, *ABCB1*, and *ABCG2* both in *in-vitro* and *in-vivo* CRC models. In addition, high expression of *STIL* mRNA was found to be associated with reduced disease-free survival in CRC cases. Interestingly, we observed that *STIL*-mediated regulation of stemness and drug resistant genes is not exclusively governed by Sonic hedgehog (Shh) signaling. Remarkably, we found *STIL* regulate β-catenin levels through p-AKT, independent of Shh pathway. This partially answers Shh independent regulatory mechanism of cancer stem cell (CSC) markers by *STIL*. Our study suggests an instrumental role of *STIL* in molecular manifestation of CRC and progression.

## Introduction

*SCL/TAL1 interrupting locus* (*STIL*) gene is a crucial factor in centriole biogenesis, and dysfunction of this gene has been associated with abnormal brain development leading to microcephaly (1). Being a 1288 amino acid cytoplasmic protein, *STIL* functions as a cell cycle-regulatory protein specifically recruited at the mitotic centrosome to promote the duplication of centrioles in dividing cells. *STIL* has been shown to interact with CDK1, PLK4, and SAS-6,which are crucial in centriole duplication (2) and thus proven to have a crucial role in cell division (3). In lung cancer, upregulation of *STIL* has been reported to have significant effect on tumor mitotic activity (4). *STIL* also has been reported to be overexpressed in pancreatic ductal cell carcinoma (5) and altered in leukemia (6). Further, *STIL* overexpression has been reported to cause chromosomal instability in cancer cells (7). *STIL* has been studied as an integral part of Sonic hedgehog signaling (Shh) cascade. The C terminus of *STIL* can interact with conserved components of the Shh signaling such as suppressor-of-fused homolog (SUFU) and GLI1. Interaction of *STIL* with SUFU inhibits the repressor function of SUFU towards GLI1, resulting in activation of Shh-GLI1 cascades. Knockdown of *STIL* has been shown to increase nuclear accumulation of SUFU with GLI1 and repression of GLI1 mediated transcriptional activity (5). *STIL* ^−/−^ mouse embryos have been shown to have reduced expression of *PTCH1* and *GLI1* and have been reported to lack primary cilia, a structure present in almost all cell types mediating Shh signaling (8, 9). During the process of carcinogenesis, increased expression of *STIL* promotes the transcriptional activity of GLI1, leading to increased transcription of GLI1 targets that promote sustained proliferation, cell death resistance, stemness, angiogenesis, and genomic instability, which are the hallmarks of cancer (10). Therefore, increase in *STIL* expression likely represents a crucial step toward cancer progression. *STIL* depletion has been shown to enhance DNA double-strand breaks caused by DNA damaging agents in ovarian cancer (11). Another report has shown *STIL* to play a Shh-dependent role in drug susceptibility in PC12 cells (12). Further Shh signaling has been studied for its role in maintenance of CSC, metastasis, and disease recurrence in colorectal cancer (CRC) (13). *GLI1*-mediated Shh signaling has been found to have important role in CRC cell survival upon therapeutic insults (14). However, there have been hardly any studies deciphering the role of *STIL* in CRC tumorigenesis and drug resistance. Studies have shown regulation of ABCB1 and ABCG2 efflux pumps to be Shh dependent, which suggest that the Shh pathway could be a potent target to overcome drug resistance and surge chemotherapeutic response (15). Nevertheless, tumor fate against therapies is governed by various cues from tumor microenvironment, and thus a complex network of inter- and intracellular signaling decides therapy response. There has been reports suggesting the instrumental role of Wnt-Shh cross talk in basal cell carcinoma (16) and gastric cancer (17). In addition, cross talk between Wnt and Shh has been shown to be contributing towards CRC progression (18); however, core pathway components mediating cross talk still remain unexplored. Further studies to explore the mediators of this cross talk would provide an in-depth mechanistic perspective on tumor development and therapy resistance in CRC. In this study, we have explored the multifaceted role of *STIL* in CRC and also deciphered its role in mediating cross talk between Shh and Wnt signaling.

## Methods

### Patients’ Samples

Biopsies were collected from CRC patients undergoing curative surgery between 2014 and 2017 at the Regional Cancer Centre, Trivandrum, after human ethics committee approval and sanction from the Institutional Review Board*. All subjects gave written informed consent in accordance with the Declaration of Helsinki.* Patients’ details and clinical information were collected from medical records of the same institution.

### Cell Culture

HCT116, HT-29, and HEK293T cells (ATCC, USA) were cultured with Dulbecco’s Eagle’s Medium (DMEM) (Invitrogen, USA) supplemented with 10% FBS (Invitrogen, USA) at 37°C and 5% CO_2_ in a humidified incubator (Thermo scientific, USA).

### Chemical Inhibition of Shh Signaling

SANT1 [(4-Benzyl-piperazin-1-yl)-(3,5-dimethyl-1-phenyl-1H-pyrazol-4-ylmethylene)-amine], a chemical inhibitor of SMO receptor, was used at a concentration of 30 nM for 24 h to inhibit Shh signaling in HT29 cells. Post treatment, cells were processed for RNA and protein isolation.

### RNA Isolation

Trizol reagent (Invitrogen, USA) was used to isolate total RNA from around 3×10^6^ adherent cells or 50 mg tissues following manufacturer’s protocol. RNA quality and quantification were carried out on Nanodrop 1000 (Thermo Scientific, USA) after assessing its quality using gel electrophoresis.

### Real-Time q-PCR

One µg of RNA was used for conversion of cDNA from each sample using PrimerScript cDNA conversion kit (TAKARA, Japan), following manufacturer’s protocol. Quantitative real-time PCR was performed using SYBR-Green-based fluorescence detection kit (TAKARA, Japan) and HT9700 detection system (AB, Life science, USA). Twenty-five ng of cDNA was used as template for each reaction. Analyzed genes and the primers used are shown in **Supplementary Table 1**. PCR data were analyzed using Data assist software (AB, Life science, USA).

### Lentiviral-Mediated Gene Silencing

HEK293T cells (3×10^5^) were seeded in a six-well plate (Nunc, Thermo, USA) with DMEM media (Invitrogen, USA) containing 10% FBS (Invitrogen, USA) and incubated till it reached 60% confluency. DNA constructs were mixed in a fixed ratio in a vial containing 75 µl OptiMEM media (Invitrogen, USA) as shRNA (0.75 µg/well), pREV (0.5 µg/well), pMDL (0.18 µg/well), and pVSVG (0.26 µg/well) (packaging plasmids were gifted from Vinay Tergaonkar, Department of Biochemistry, Yong Loo Lin School of Medicine, NUS). Details of shRNA employed are given in **Supplementary Table 2**. To the mixture of constructs, 2 mg/ml polyethylenimine (PEI) (Sigma-Aldrich, USA) was added followed by brisk vortex for 10 s and incubated for 30 min at RT. During incubation, HEK293T cells were rinsed with OptiMEM media, and 688 µl of OptiMEM was added to each well. After incubation, 75 µl DNA/PEI complex was added to cells drop-wise followed by gently shaking, and cells were kept in incubator overnight. Fresh 1.5 ml DMEM media with 10% FBS was added to the cells after overnight incubation, and media was collected every 24 h from cells and replenished by fresh media for 3 consecutive days. Collected media was filtered using 0.45 µm PES membrane (Minisart, Sartorius, Germany) to remove extracellular debris. Filtered media containing lentiviral particles were centrifuged at 24,000 rpm at 20°C for 2 h in 90Ti rotor in ultracentrifuge (Beckman Coulter Optima TM L-100K). Viral particle pellet was obtained after discarding supernatant post centrifugation and suspended in 100 µl OptiMEM media overnight at 4°C.The collected lentivirus suspension was used for transfection or stored for later use at −80°C. Then 3 × 10^5^ number of HT29 and HCT116 cells were seeded in six-well plate in DMEM media containing 10% FBS and incubated till it reached 70% confluency. After reaching desired confluency, cells were infected with 100 µl of lentiviral particle suspension along with 10 µg/ml polybrene (Sigma-Aldrich, USA) and 1.5 ml of fresh DMEM media containing 10% FBS without antibiotics and incubated for a minimum of 24 h. Cells were selected using puromycin (Sigma-Aldrich, USA) antibiotic with a concentration of 2 µg/ml and 7.5 µg/ml for HCT116 and HT29, respectively, for 3 weeks for the development of stable transfected cells. Gene knockdown was evaluated using real-time qPCR and western blot.

### Cell Proliferation Assay

MTT-3-(4, 5-Dimethylthiazol-2-yl)-2, 5-Diphenyltetrazolium Bromide) assay was used to determine cell survival. One hundred μl of culture medium containing 1×10^6^ cells were seeded into a 96-well plate and incubated for 24 h. After that 0.5 mg/ml of MTT was added to each well and incubated for 3 h in the dark at 37°C. Culture medium from the wells was aspirated, and 100 µl of isopropanol was added to dissolve the formazan crystals formed inside cells. Absorbance was measured at 570 nm in a microtiter plate reader. A graph was plotted with concentration on the X axis and absorbance on the Y axis.

### Cell Cycle Analysis

Cells were harvested after trypsinization by centrifugation at 1,200 g for 5 min at 4°C. Then 300 µl of ice-cold PBS was used for the suspension of cells, and 700 µl of 70% ethanol was added drop-wise with slight vortex followed by incubation for 1 h in ice. Post incubation, cells were centrifuged at 1,200 g for 5 min at 4°C, and the cell pellet was suspended in 1× PBS. Cells were treated with RNase A (Sigma-Aldrich, USA) at a concentration of 100 µg/ml for 1 h at 37°C in a heat block (Thermo mixer, Eppendorf, Germany). After incubation, 10 µg/ml of propidium iodide was added in the dark for 15 min. Cells were strained using 45 µm cell strainer and collected in FACS tube. Tubes were kept in ice till flow analysis.

### Immunophenotyping

Cells were harvested after trypsinization by centrifugation at 400 g for 5 min and washed with 1 ml HBSS. Then 1×10^6^ cells were used for antibody staining according to manufacturer’s protocol. Details of antibodies used are provided in **Supplementary Table 3**. After antibody staining, cells were washed with ice-cold HBSS and suspended in HBSS containing 2% FBS. Cells were kept on ice till flow cytometry analysis was carried out using BD FACS ARIA II (BD Bioscience, USA).

### Side Population Analysis

Side population assay was performed according to the protocol developed by Goodell (1996) with slight modifications. Harvested cells were spun at 400 g for 5 min, and pellet was suspended at 1×10^6^ cells per ml in prewarmed DMEM (supplemented with 2% serum) media (Gibco, Invitrogen) in two tubes. Hoechst 33342 (Sigma-Aldrich) was added to a final concentration of 5 µg/ml at dark in both tubes. In control tube, 100 µm verapamil was added to cells prior to addition of 5 µg/ml Hoechst. Cells were mixed well and placed in 37°C dry bath (Thermomixer, Eppendorf, Germany) with shaking at 500 rpm. After incubation, cells were spun down at 4°C and resuspended in cold HBSS (with 2% serum). Cells were suspended in ice-cold HBSS (2% serum) containing 2 µg/ml Propidium iodide (Sigma-Aldrich, USA) for dead cell discrimination. Cells were immediately taken for flow cytometry analysis (BD FACS Aria II), and results were analyzed using FACS Diva software.

### Apoptosis Assay

Cells were seeded in a six-well plate and incubated for 24 h. Then 5-Flurouracil was added to wells at a sublethal concentration, keeping DMSO treatment as control for 24 h. Cells were washed with 1× PBS followed by trypsinization and pelleted by centrifugation at 400 g for 3 min at 4°C. Cells 100 µl were suspended in 1× binding buffer and 5 µl of Annexin reagent and incubated in the dark for 15 min at RT. After incubation, 5 µl propidium iodide was added to cells and kept for 5 min. Finally, 400 µl of 1× binding buffer was added, and cells were filtered using 40 µm strainer and analyzed by flow cytometry using BD FACS Aria II (BD Bioscience, USA).

### Protein Isolation

The cells were washed with 1 ml 1× PBS. Then 1 ml 1× PBS was added, and the cells were scraped out using a cell scraper. The cells were collected into a vial and centrifuged at 5,000 rpm for 5 min at 4°C. The supernatant was discarded, and 50–200 µl of RIPA buffer was added based on the pellet size. Phosphatase and Protease inhibitors (10 µl/1 ml buffer) were added. The pellet was suspended well and vortexed and further mixed in thermomixer at 4°C for 1.40 h at 1,400 rpm and centrifuged at 14,000 rpm for 15 min at 4°C. The supernatant was collected and stored at −80°C.

### Nuclear and Cytoplasmic Protein Fraction Isolation

Approximately 1 × 10^7^ cells were washed with 1× PBS and harvested by centrifugation at 400 g for 5 min. The pellet was resuspended in 5 pellet volume of CE buffer (1× solution composed of 10 mM HEPES, 60 mM KCl, 1 mM EDTA, 0.075% (v/v) NP-40, 1 mM DTT, and 1 mM PMSF, pH 7.6), incubated on ice for 3 min, and spun at 1,500 rpm for 4 min. The supernatant (cytoplasmic extract) was removed into a clean tube, and the pellet was washed with 100 μl of CE buffer without NP-40 at 1,500 rpm for 4 min. One pellet volume of NE buffer (1× solution composed of 20 mM Tris Cl, 420 mM NaCl, 1.5 mM MgCl2, 0.2 mM EDTA, 1 mM PMSF, and 25% (v/v) glycerol, pH 8.0) was added to the pellet, salt concentration adjusted to 400 mM using 5 M NaCl (~35 μl), and an additional pellet volume of NE buffer added to it and resuspended by vortexing. It was incubated on ice for 10 min with periodic vortexing. Both CE and NE were then spun at maximum speed (14,000 rpm) for 10 min. The supernatants from all the tubes were then transferred separately to clean tubes and stored at −80°C.

### Western Blot

After SDS-PAGE separation of proteins, transfer sandwich was prepared with PVDF membrane and placed in transfer buffer. PVDF membrane (Amersham) was activated with methanol for 15 s prior to use. The blot was allowed to run for 120 min at 100 volts. After the transfer of proteins, membrane was blocked using 5% skimmed milk powder as blocking buffer for 1 h at RT. Then membrane was washed with 1× TBST buffer and incubated with primary antibody overnight (details of antibody in **Supplementary Table 3** **).** Again, membrane was washed in 1× TBST buffer followed by secondary antibody (1:5,000 dilution) treatment, then incubated for 1 h at RT. After incubation, membrane was washed with 1× TBST buffer three times and detected with ECL reagent (TAKARA, Japan) using Versa Doc (BD Bioscience, USA) or X-ray film.

### Development of Tumor Xenograft

Each experimental groups had five 8-week-old male NOD/SCID mice, which were housed in a specific pathogen-free environment. Four million cells/mice of each control shRNA and STIL-shRNA HT-29 cells were suspended in PBS and injected into flanks subcutaneously. Then mice were monitored every 2 days for tumor growth and health. Once visible tumor appeared, its volume was measured using calipers every 2 days for obtaining rate of tumor growth in both groups. Tumor volume was calculated using the formula V (mm^3^) = (Largest length) × (Shortest length)^2/^1 (19). At the end, mice were sacrificed using carbon dioxide inhalation, and tumors along with lungs and liver tissues were harvested and stored in RNAlater and 4% paraformaldehyde for molecular and histological analysis, respectively.

### H&E Staining

Hematoxylin and Eosin (Merck-Millipore, USA) staining was performed according to manufacturer’s protocol for pathological examination of various tissues obtained after surgery.

### Immunohistochemistry

Tissues received post-surgery were fixed with 4% paraformaldehyde (Sigma-Aldrich, USA) at 4°C for 16 h. They were then processed with alcohol gradient and xylene followed by embedding with paraffin to make paraffin blocks. Five µm thick sections were mounted on Starfrost glass slides (Leica, Germany) and were deparaffinized in xylene followed by rehydration with alcohol gradients. Sections were blocked for endogenous peroxidases, and antigen retrieval was performed using 10 mM citrate buffer (pH 6.0). After retrieval, BSA (3%) blocking was done for 30 min at RT followed by primary antibody staining overnight. Primary antibody details are given in **Supplementary Table 3**. After incubation, antigen-antibody binding was detected using ABC kit (Vecta stain, USA). DAB substrate (Sigma-Aldrich, USA) was used as a chromogen, and hematoxylin (Merck Millipore, Germany) was used as a counterstain. Sections without incubation of primary antibody served as negative control. Semiquantitative analysis was done by counting three independent microscopic fields (n=100–200 cells/field) for staining of specific antigen using upright microscope (Leica DM1000, Germany). Tissue array (US Biomax, USA) for *STIL* expression was also performed according to above protocol. Tissue array demographics has been detailed in **Supplementary Table 4**.

### Cancer Datasets Analysis

*STIL* gene was used as an input in Oncomine **(** www.oncomine.org/resource/) and GEPIA (http://gepia.cancer-pku.cn/) online free cancer database to obtain gene expression pattern across multiple colorectal cancer transcriptomics studies globally, and survival curves for *STIL*-expressing patients were obtained from C-Bioportal (www.cbioportal.org) online tool.

### Statistical Analysis

All statistical analysis was performed using SPSS version 25 and GraphPad Prism 5. Unpaired t-test was performed for mean comparison and p value determination for flow cytometry and immunohistochemistry data. Real-time PCR data analysis was carried out using one sample t-test to compare determined log_2_ fold change and determined p value among samples. Kaplan-Meier plots were obtained from C-Bioportal cancer genomic datasets. Multivariate analysis was performed using binary logistic regression model, and p-value and ODDS ratio were derived.

## Results

### Elevated Expression of *STIL* Gene in Tumors Correlates With Lower Disease-Free Survival In CRC Patients

**Figure 1:**
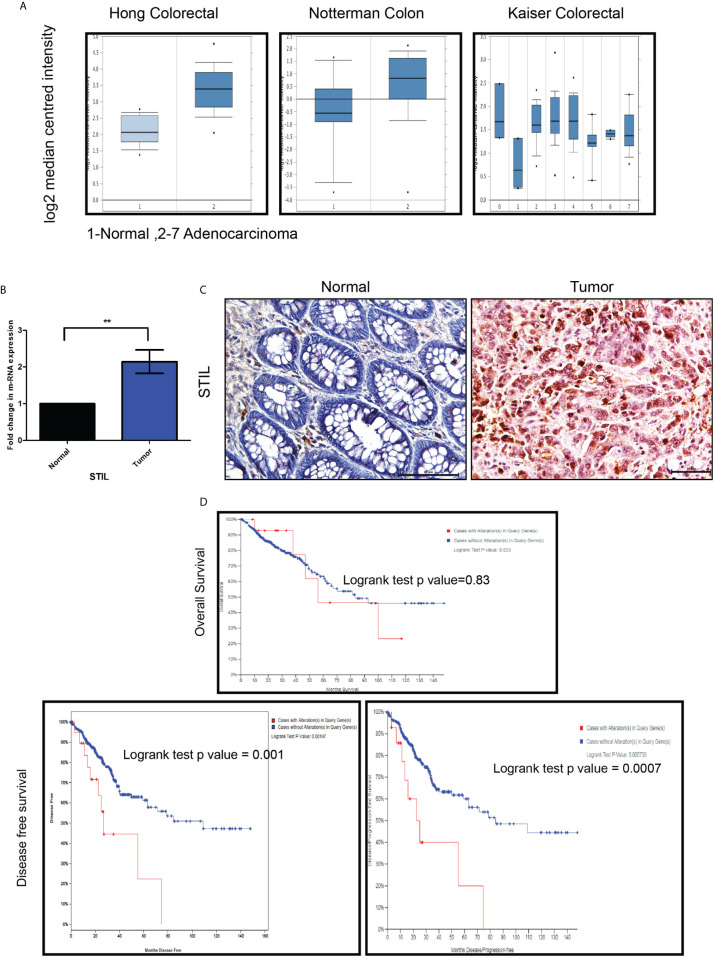
Expression of STIL gene in CRC and its role in prognosis. **(A)** STIL m-RNA expression in normal colon and tumor obtained from Oncomine datasets. **(B, C)** Differential expression of STIL gene mRNA (N=22) and protein in CRC. **(D)** Kaplan-Meier plot showing association of high mRNA expression with patients’ survival and disease free survival in CRC cases. One Sample t-test and log rank test results showing p value ≤0.01 is represented by **, Scale bar is 20µm. Note: In Kaplan-Meier plot, red and blue line in indicates high and low m-RNA expression respectively.

We used Oncomine datasets to analyze differential mRNA expression of *STIL* between normal and cancerous colon tissues from three different studies (Hong, Notterman, and Kaiser), which revealed a significant enrichment of *STIL* gene in cancer tissues (**Figure 1A**). Similar trend in *STIL* mRNA expression was observed in TCGA datasets, using GEPIA web tool (20) (**Supplementary Figure 1A**). Further validation in our cohort CRC using real-time q-PCR showed a 2.1-fold high expression in tumor compared to matched normal tissues (p = 0.001) (**Figure 1B**). IHC of *STIL* protein in cancer tissues showed a very high cytoplasm-specific staining compared to normal tissue (**Figure 1C**). To assess the role of *STIL* enrichment in colon cancer with disease prognosis, we used c-Bioportal cancer database. Kaplan-Meier plots showed no significant differences for 5-year survival among *STIL* high- and low-expression groups; however, *STIL* high-expression group showed a very low disease-free survival rate among CRC patients with a p value 0.001 and 0.0007 in two different studies (**Figure 1D**). Put together, these results suggest that overexpression of *STIL* in CRC could have crucial unexplored role in tumor development and disease progression.

### High *STIL* Protein Expression Is Found to Have Association With Early Tumor Stage in Rectal Cancer

**Figure 2:**
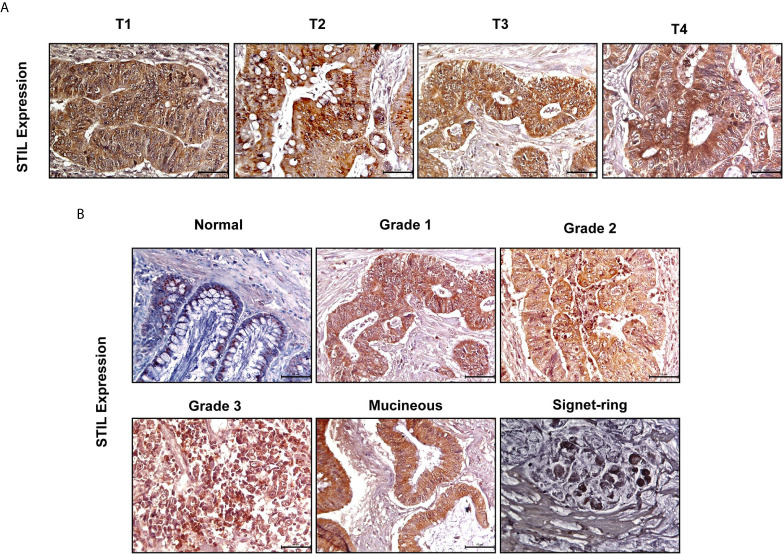
IHC of rectal cancer tissue array showing expression of *STIL* protein. **(A, B)** Representative images showing expression of *STIL* protein in different tumor stages and grades.

To evaluate STIL protein expression in different tumor stages and grades of CRC, we performed IHC for *STIL* in rectal cancer tissue array (array details given in **Supplementary Table 4**). *STIL* was found to have intense cytoplasmic staining in cancer tissues compared to normal (p value 0.001). Early-stage (T1-T2) tumors were stained positively with a high IHC score compared to late-stage (T3-T4) tumors. (**Figure 2A** and **Table 1**). Both high- and low-grade tumor tissues showed moderate to strong *STIL* staining except signet-ring carcinoma (**Figure 2B**). Chi-square analysis revealed that females (69.4%) and early-stage tumor (73.3%) correlated well with higher expression of *STIL* with a p value of 0.04 and 0.03, respectively (**Table 1**). However, sex-adjusted binary regression analysis revealed that higher tumor stage has 71% lesser chance of high expression of *STIL* compared to early stage (p value 0.02), and earlier association of female sex with *STIL* expression was influenced by tumor stage. In addition, late-disease stage was found to have 60% lesser chance of *STIL* expression compared to early stage (p value 0.07) (**Table 2**). In addition, role of *STIL* in cancer cell migration was evaluated using scratch wound healing assay, which showed no significant difference between control and *STIL*-silenced HCT116 cells (**Supplementary Figure 2**). Further, we also analyzed lung and liver tissues for presence of secondary tumor in control and *STIL*-silenced xenograft mice using Ep-CAM protein expression and found no tumor deposits (**Supplementary Figure 3**). This further suggests that *STIL* may not play a role in cancer cell migration and thus metastasis in concordance to tissue array observation. Put together, this study suggests that *STIL* may not play a significant role in invasion of tumor; however, it could have a crucial role in early tumorigenesis.

**Table 1:** Association of clinical and pathological parameters with *STIL* protein expression.

		IHC score		Total %	P- value
		Low%	High%		
	Female	(11) 30.56	(25) 69.44	(36) 100	0.04
	Male	(21) 53.85	(18) 46.15	(39) 100	
**Age (Years)**	≤55	(17) 45.95	(20) 54.05	(37) 100	0.57
	>55	(15) 39.57	(23) 60.53	(38) 100	
**Pathology**	Normal	(3) 60	(2) 40	(5) 100	0.001
	Carcinoma	(21) 33.87	(41) 66.13	(62) 100	
	Others	(8) 100	0	(8) 100	
**Tumor Stage**	≤T2	(8) 26.67	(22) 73.33	(30) 100	0.03
	>T2	(21) 52.50	(19) 47.50	(40) 100	
**Disease Stage**	I	(8) 28.57	(20) 71.43	(28) 100	0.07
	II & III	(21) 50	(21) 50	(22) 100	

**Table 2:** Association of tumor and disease stage with *STIL* expression.

		ODDs Ratio	95% CI	P- value
**Gender**	Female	1	0.13-1.05	0.06
	Male	0.37		
**Tumor Stage**	≤T2	1	0.10-0.84	0.02
	>T2	0.29		
**Disease Stage**	I	1	0.14-1.10	0.07
	II & III	0.4		

### *STIL* Silencing Reduced Cell Proliferation and Tumor Growth in CRC

**Figure 3:**
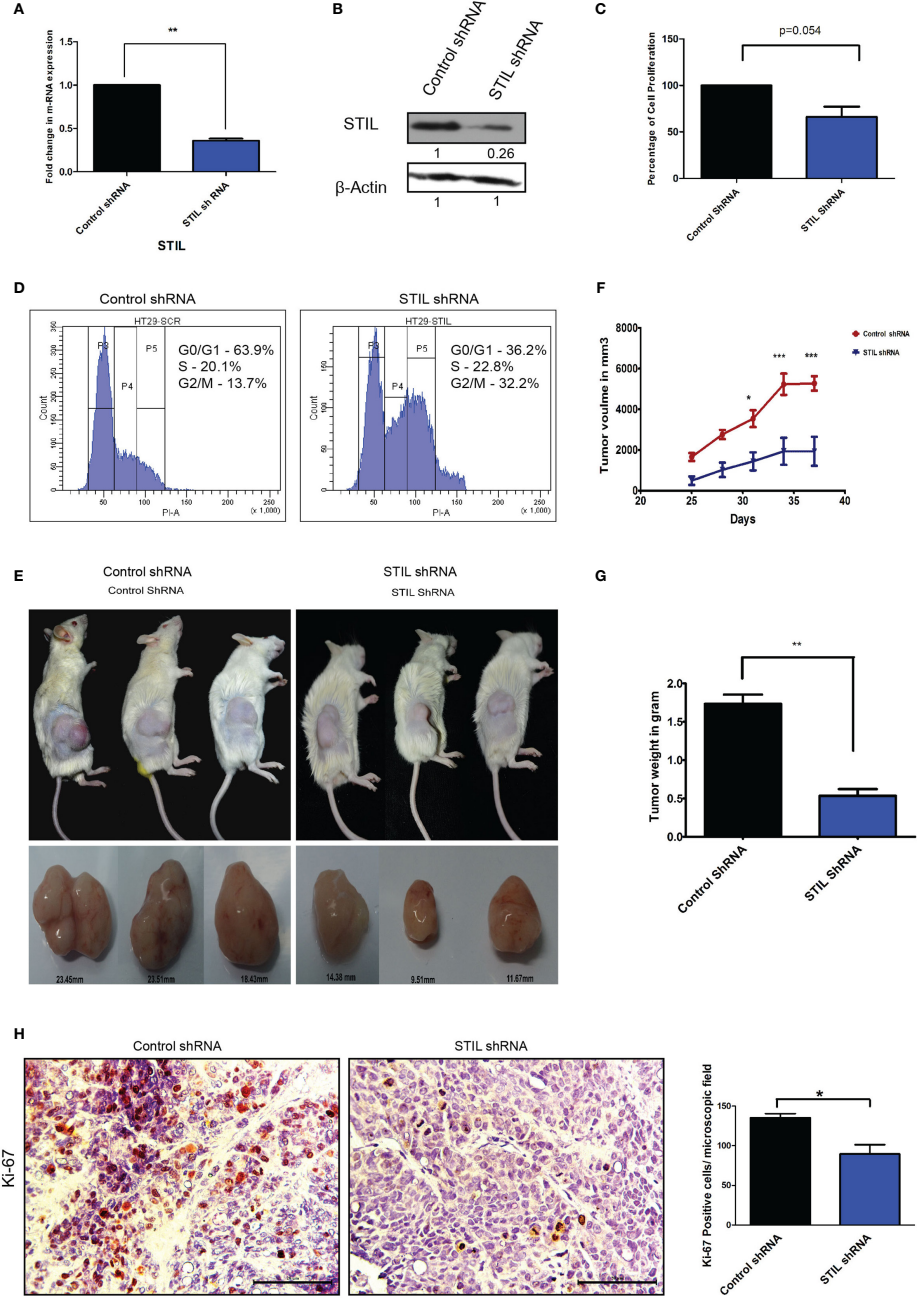
Role of *STIL* in CRC tumorigenesis. **(A)** Bar graph showing fold change in STIL mRNA expression upon STIL silencing. **(B)** Immunoblot validating *STIL* silencing at protein level. **(C)** Bar graph showing cell proliferation of HT-29 cells upon *STIL* silencing. **(D)** Histogram showing effect of *STIL* silencing on cell cycle. **(E, F)** Representative images of tumor xenograft and graph showing growth of tumor upon time (n = 3). **(G)** Bar graph showing mean dry weight of tumor in both groups (n = 3). **(H)** IHC showing nuclear expression and quantification of Ki-67 protein in xenograft derived tumor tissues. Unpaired t-test results showing p value ≤ 0.05, ≤ 0.01, ≤ 0.001 are represented by *, **, ***, respectively, Scale bar is 20 µm.

To understand the role of *STIL* in proliferation and tumor growth of CRC, we stably silenced *STIL* gene in HT-29 CRC cells using shRNA (**Figures 3A, B** **)**. Cell proliferation was evaluated by MTT assay, where *STIL* silencing reduced the proliferation by 34% compared to control shRNA cells (p=0.05) (**Figure 3C**). *STIL* has been well studied for its role in cell cycle regulation, as it plays a crucial role in centrosome assembly. Thus, we analyzed cell cycle pattern, which revealed a two-fold accumulation of cells in G2/M phase in *STIL*-silenced cells (**Figure 3D**). These results led us to investigate the tumorigenic potential of *STIL in-vivo*, for which we developed a tumor xenograft using *STIL*-silenced HT-29 cells and compared it with control shRNA xenograft. We observed a higher tumor volume in control group, which became more significant with time compared to *STIL*-silenced tumor (**Figures 3E, F** **)**. Dry weight of control tumor was found to be threefold more than *STIL*-silenced tumor (p=0.001) (**Figure 3G**). Further, we analyzed nuclear expression of the proliferation marker Ki-67 in tumor xenograft tissue and found a significantly reduced expression in *STIL*-silenced group compared to control (p=0.01) (**Figure 3H**). These results confirm a significant role of *STIL* in regulating tumor proliferation and growth in CRC.

### *STIL* Regulates Expression of Cancer Stem Cell–Associated Genes in CRC Independent of Shh Signaling

**Figure 4:**
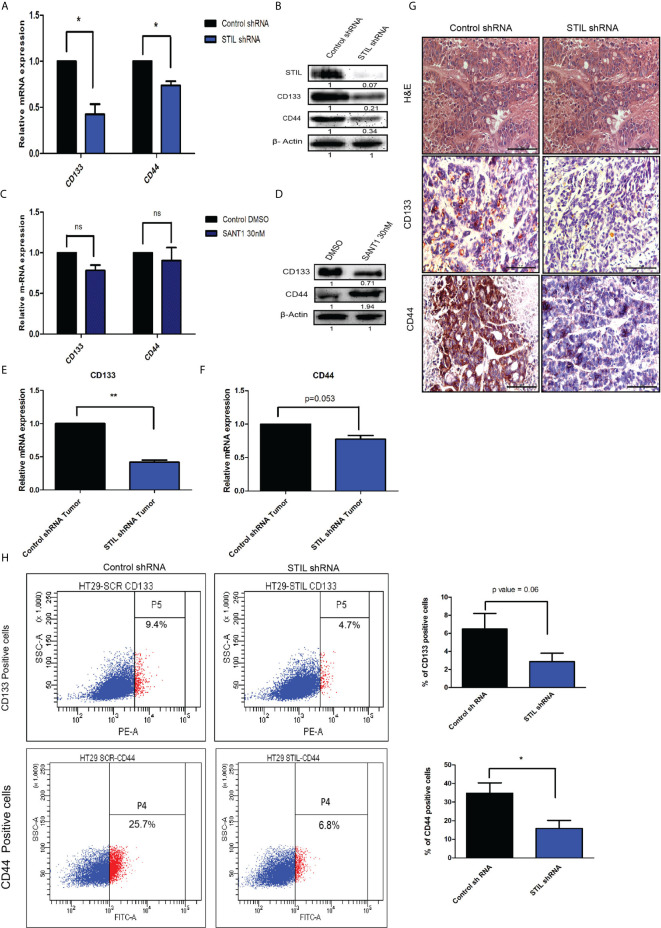
Effect of *STIL* silencing on CSC markers expression in CRC. **(A, B)** mRNA and protein level expression of CSC markers upon *STIL* silencing. **(C, D)** mRNA and protein level expression of CSC markers upon SANT1 treatment. **(E–G)** Showing *CD133* and *CD44* expression in xenograft tumors at mRNA and protein levels, respectively. **(H)** Dot plot representing % of *CD133* and *CD44*-positive cells. Unpaired t-test results showing p value ≤ 0.05, ≤ 0.01, ≤ 0.001 are represented by *, **, ***, respectively. Scale bar is 20 µm.

We have observed *STIL* overexpression to be associated with lower disease-free survival in CRC patients from our initial results. Disease recurrence has mostly been contributed by residual cells, which are drug resistant in nature. These cells are known to have cancer stem cell (CSC) properties including expression of established surface markers. However, the role of *STIL* in CRC stem cells is still unknown. We assessed the expression of established CSC markers in *STIL*-silenced cells and observed a significant reduction in *CD133* (fold change 0.4, p=0.03) and *CD44* (fold change0.7, p=0.02) expression in mRNA and protein (**Figures 4A, B** **)**. *STIL* is a known positive regulator of Shh signaling, and thus, we analyzed expression of CSC markers *CD133* and *CD44* upon Shh inhibition by SANT1 treatment and found no significant reduction in their mRNA levels. Interestingly, *CD44* protein expression was found elevated, suggesting *STIL*-mediated regulation of CSC markers may not be exclusively through Shh signaling (**Figures 4C, D** **)**. We further validated these findings in xenograft tumor tissues using real-time q-PCR, which showed significant reduction in *CD133* (fold change 0.41, p=0.002) and *CD44* (fold change 0.77, p=0.05) mRNA levels in *STIL*-silenced tumor, which was further confirmed by IHC (**Figures 4E–G**), respectively. To examine if this stands true across other CRC cells, we analyzed *CD133* and *CD44* expression in HCT116 cells upon *STIL* silencing and SANT1 treatment, which also showed similar trend (**Supplementary Figures 4A–E**). Additionally, we also analyzed expression of stem cell maintenance factors *oct4* and *nanog* in *STIL*-silenced cells and found a reduction (fold change 0.69 and 0.78) compared to control, respectively; however, statistical significance was not observed (**Supplementary Figures 4F–H**). Membranous expression of *CD133* and *CD44* are known to be characteristics of CSC; thus, we also analyzed their surface expression using flow cytometry. In concordance with our gene expression data, a significant reduction in *CD133* and *CD44*-positive cells were also observed upon *STIL* silencing (**Figure 4H**). These results confirm a *STIL*-mediated regulation of CSC signatures associated with CRC.

### *STIL* Silencing Reduced Drug Efflux Activity *Via* Depletion Of Drug Transporter Proteins in CRC Cells

**Figure 5:**
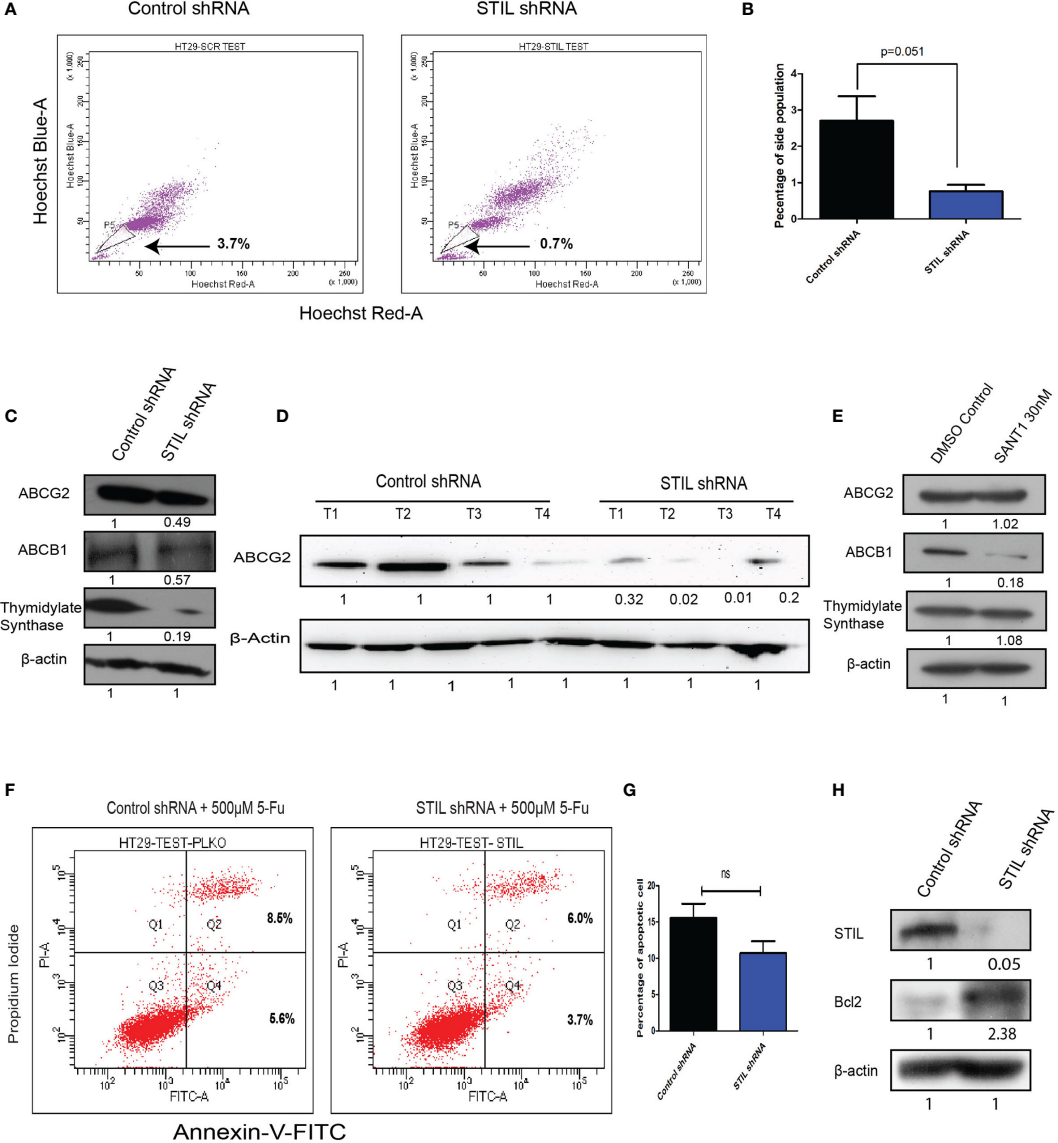
Effect of *STIL* silencing on drug effluxing cells and associated genes in CRC. **(A, B)** Dot plot showing side population cells and bar plot representing quantification of the same. **(C)** Western blot showing expression of ABCG2, ABCB1, and thymidylate synthase protein expression, upon *STIL* silencing. **(D)** Western blot showing ABCG2 protein expression in control and *STIL*-silenced tumor xenograft. **(E)** Western blot showing expression of ABCG2, ABCB1, and thymidylate synthase protein expression, upon SANT1 treatment. **(F, G)** Annexin-V assay showing percentage of cell death upon 5-FU treatment. **(H)** Western blot showing Bcl2 expression upon STIL silencing. Unpaired t-test results showing p value ≥ 0.05, is represented by ns, not significant.

Another hallmark of CSC is the ability to efflux drugs *via* active expression of drug transporter proteins. Here, we performed side population (SP) assay, which potentially identifies drug effluxing cells and CSCs. *STIL* silencing resulted in reduced SP in HT-29 cells with mean % population of 0.7 compared to control with 3.7 (p=0.051) (**Figures 5A, B** **)**. We noted similar reduction in SP in HCT116 cells upon *STIL* silencing (**Supplementary Figure 5A**). Moreover, *ABCG2*, which is known to be the crucial transporter associated with stem cells and SP, was found to have reduced levels of mRNA and protein in both *STIL*-silenced HT29 cells and xenograft tumor tissue (**Supplementary Figures 5B, C** and **Figures 5C, D** **)**. Our data further revealed depletion of *ABCB1* and *ABCG2* protein upon *STIL* silencing in HT29 cells and HCT 116 cells **(** **Figure 5C**, **Supplementary Figure 5D**). In addition, *STIL* silencing was also found to deplete expression of *thymidylate synthase* (*TS*) enzyme, known for its role in 5-fu resistance in CRC (**Figure 5C** and **Supplementary Figure 5D**). However, we observed no reduction in ABCG2 and TS protein expression upon SANT1 treatment, revealing a Shh-independent regulation of these proteins by *STIL* (**Figure 5E** and **Supplementary Figure 5E** **)**. However, *ABCB1* expression reduced significantly upon Shh inhibition (**Figure 5E**). Further, we analyzed the effect of *STIL* silencing on 5-fu drug treatment in HT29 cells. To our surprise, a slightly reduced cell death in *STIL*-silenced cells was observed compared to controls (**Figures 5F, G** **)**. To investigate the cause behind reduced cell death, we looked for expression of antiapoptotic protein Bcl2 upon *STIL* silencing and observed an increased Bcl2 protein levels compared to control (**Figure 5H**). Put together, these data suggest that *STIL* may regulate drug efflux and resistance markers not solely through Shh pathway in CRC. However, the role of *STIL* in 5-fu sensitization for CRC cells demands further investigation.

### *STIL* Regulates *β-Catenin* Expression Seemingly *via AKT* Contrarily to Shh Signaling

**Figure 6:**
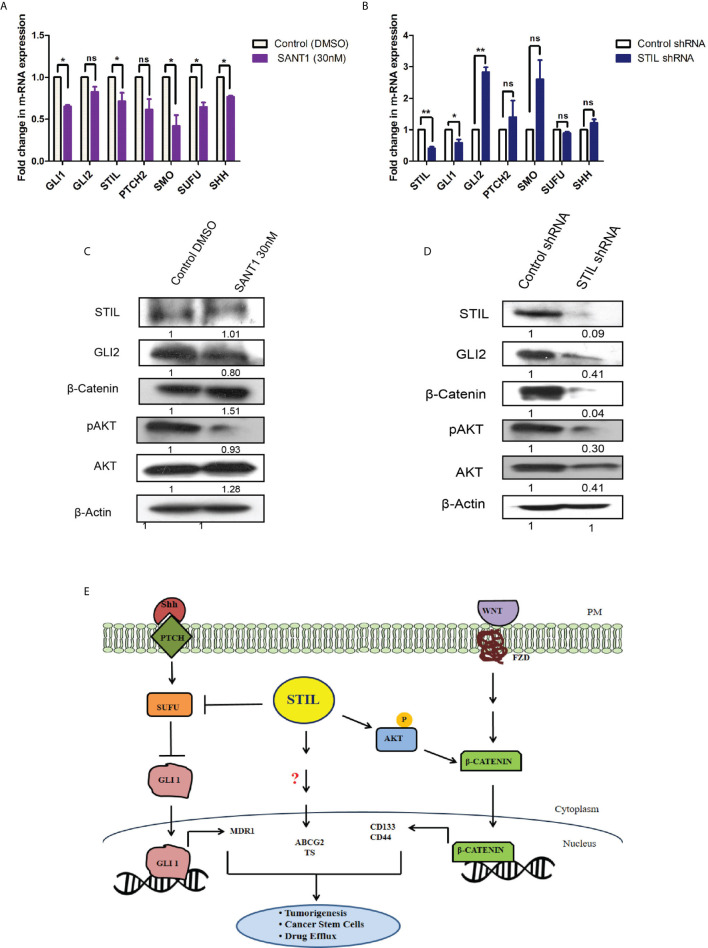
Effect of SANT1 treatment and *STIL* inhibition on Shh and Wnt signaling. **(A, B)** Bar graph showing expression of Shh signaling components upon SANT1 treatment and *STIL* silencing. **(C, D)** Western blot showing effect of SANT1 treatment and *STIL* silencing on GLI2, β-catenin, AKT, and p-AKT protein expression. **(E)** Schematic representation showing possible mechanistic regulation of multifaceted role by *STIL* in CRC. Unpaired t-test results showing p value ≤ 0.05, ≤ 0.01 are represented by *, **, respectively; ns, not significant.

*STIL* has been studied to be a positive regulator of hedgehog signaling; however, *STIL*-mediated hedgehog regulation in CRC remains unknown. To study its role in mediating regulation of hedgehog signaling in CRC, we screened for expression of hedgehog-associated genes (*SHH, PTCH2, SMO, SUFU*, and *STIL*) as well as effector genes (*GLI1* and *GLI2*) after *STIL* silencing and treatment with SANT1. SANT1 treatment resulted in significant downregulation of the pathway-associated genes as well as effectors molecules *GLI1&2* (**Figure 6A**). Conversely, *STIL* repression did not show similar trend in gene expression except for *GLI1* (**Figure 6B**). These observations suggest the negative regulatory role of *STIL* on *SUFU*, *SMO*, and *SHH* genes in CRC. Although *GLI2* showed an increase in mRNA level upon *STIL* silencing, it showed a significant reduction at protein level upon Shh inhibition and *STIL* repression (**Figures 6C, D** **)**. There are studies that advocate a reciprocal relationship between Wnt and Shh signaling in CRC development (21). We further looked into probable *STIL*-mediated cross talk between Shh and Wnt signaling and observed *STIL*-mediated regulation of Wnt effector *β-catenin*, which was not altered upon Shh inhibition (**Figures 6C, D** and **Supplementary Figures 6A, B**). Further, when we checked the cytoplasmic and nuclear *β-catenin* levels upon *STIL* silencing, we observed a reduction in cytoplasmic as well as nuclear cleaved fragment (~56 kDa), which is reported to be the transcriptionally active fragment in CRC, suggestive of inhibited Wnt signaling (**Supplementary Figure 7**). However, further studies are warranted to delineate the *STIL*-β-catenin molecular axis in CRC. There has been a recent report suggesting *STIL*-mediated regulation of AKT protein in gastric cancer (22) and another one suggesting *AKT*-mediated regulation of *β-catenin* in CRC (23). Thus, we hypothesized that *STIL* regulates *β-catenin via* regulating *AKT* in CRC. *STIL* silencing resulted in a significant decrease in AKT and p-AKT protein, suggesting *STIL*-mediated *β-catenin* regulation is *via* AKT/pAKT regulation in CRC, unlike Shh signaling (**Figures 6C, D** **)**. These results suggest that *STIL* functions in a Shh-independent manner for regulation of *β-catenin* in CRC.

## Discussion

*STIL* alteration has been implicated in lymphoblastic leukemia and microcephaly (24, 25); however, the role of *STIL* in solid epithelial tumors stands least explored. Our study substantiates a novel role for *STIL* in regulation of tumor growth, drug resistance, and stem cells in CRC. *STIL* plays an instrumental role in centriole division and thus has been very critical for cell cycle progression and proliferation (26). Being a regulator of cell proliferation, its role in cancer becomes obvious, and many tumor types have been reported to have enriched expression of *STIL* (1, 4). In this study, we found an enriched expression of *STIL* at mRNA and protein level in CRC tissues, whereas adjacent normal tissue showed a basal-level expression (**Figure 1**). However, to date the role of *STIL* in CRC growth and proliferation has not been studied. *STIL*, being a critical factor in cell cycle machinery, its downregulation resulted in aberrant cell cycle and reduced proliferation in HT29 cancer cells (**Figure 3**). Further studies with *STIL*-silenced xenograft showed a remarkable difference in tumor growth in NOD/SCID mice with lesser tumor volume and weight compared to control shRNA group. Ki-67 antigen, an established proliferation marker, was also found to have less nuclear staining in *STIL*-silenced tumor (**Figure 3**). Put together, these data showed a critical role of *STIL* in CRC proliferation and growth. Another aspect of *STIL*, which has not been explored, is its role in Shh signaling-mediated stem cell maintenance in cancer. CSC are being studied for development of future therapeutic interventions in CRC as they are known to be the fuel behind therapy failure and recurrence (27). Although CSCs are well-known to be regulated by developmental signaling cascades, specific targets are lacking, which can be exploited for development of advanced therapeutics. CD133 and CD44 surface molecules have been studied well in CRC for their enriched expression in CSCs and responsible for drug resistance, recurrence, and metastasis in CRC (28–30). Our study has shown that repression of *STIL* in HT-29 cells results in a significant reduction of CD133 and CD44-positive cells (**Figure 4**). Interestingly, *STIL* was found to regulate expression of CSC markers (**Figure 4**) and regulators such as *OCT4* and *NANOG* at the transcriptional level both *in vitro* and *in vivo* (**Supplementary Figure 4**). These data suggest a stem cell regulatory function of *STIL* in CRC. However, when Shh signaling was inhibited by SANT1, we did not observe any significant reduction in mRNA levels of *CD133* and *CD44* markers (**Figure 4**), along with *OCT4* and *NANOG* genes (**Supplementary Figure 4**). Interestingly, CD44 protein level was found elevated upon SANT1 treatment, which signs a heterogenous mode of regulation of CSC markers by *STIL* may not be solely through Shh signaling in CRC. Another hallmark feature of CSCs and drug-resistant cells is the ability to efflux drugs by enhancing ABC pumps expression (31). SP cells show high ABC transporter activities, and CSCs are reported to be enriched in SP cells in multiple gastrointestinal cancer (32). Hedgehog signaling has been reported to regulate expression of ABC transporters in ovarian epithelial cancer (33), and *STIL*, being a positive regulator of *GLI1*-mediated hedgehog regulation, was found to be significantly regulating side population in CRC cells (**Figure 5**). *ABCG2* remains as one of the major efflux pumps regulating side population as well as stem cells (34), and we observed downregulation of *ABCG2* and partial reduction in *ABCB1* expression upon *STIL* repression (**Figure 5**). Further, *STIL* silencing was found to repress expression of *thymidylate synthase* (**Figure 5**), a known target for 5-fu and a poor prognostic marker in CRC (35). To our concern we did not observe reduction of ABCG2 and Thymidylate synthase protein upon Shh inhibition, suggesting a critical role of *STIL* in regulation of drug-resistant cells and genes in CRC independent of Shh signaling (**Figure 5**). Previous studies have already shown that *ABCG2* function and localization are regulated by *AKT* (36). Since we found that *STIL* modulates AKT expression (**Figure 6**), thus *STIL*-mediated regulation of *ABCG2* in CRC could possibly be *via AKT*. Surprisingly, inhibition of *STIL* failed to sensitize HT29 cells for 5-fu treatment (**Figure 5**). Further, antiapoptotic protein Bcl2 showed enriched expression upon *STIL* silencing, partially explaining the reason behind reduced apoptosis in *STIL*-repressed cells after 5-fu treatment. Again, there are reports of increased thymidylate synthase levels upon 5-fu treatment (37), which in turn could desensitize cells to 5-fu treatment. In this study, 5-fu treatment might be elevating the TS levels, thus replenishing the reduced TS caused by *STIL* silencing resulting in 5-fu resistance. However, in a recent study, *STIL* repression was shown to have a synergistic effect with DNA-damaging agents in ovarian cancer (11). This suggests that function of *STIL* in therapy sensitization could be drug and tissue specific. Nevertheless, this heterogeneous mode of action of *STIL* in 5-fu and other chemotherapeutics needs further investigation.

*STIL* has been established to be a positive regulator of Shh signaling and known to regulate *GLI1* (38). Shh inhibition by SANT1 treatment showed reduced gene expression of its pathway components including *GLI1*/2; however, *STIL* silencing was found to downregulate *GLI1* and *GLI2* but no other Shh components (**Figure 6**). These results advocate for a Shh-independent regulatory function of *STIL*, which demands further studies. Shh and Wnt signaling has been studied for their antagonistic mode of regulation (21), and Wnt being the most critical aberrant signaling in CRC (39), we further looked into *STIL*-mediated regulation of Wnt pathway. Interestingly, we found *STIL* to be regulating β-catenin protein. However, Shh inhibition did not show similar result, which signs at existence of a *STIL*-mediated molecular axis between Wnt and Shh signaling (**Figure 6**). A recent study has delineated that *STIL* regulates *PI3K/AKT* in gastric cancer. Reiteratively, AKT is known to regulate β-catenin stabilization (40, 41). These studies led us to explore an *AKT* mediated *β-catenin* regulation by *STIL*. One of the most interesting findings of our study remains to be *STIL*-mediated regulation of *β-catenin via* AKT and p-AKT independent of Shh signaling (**Figure 6**). Eventually, our study revealed a *STIL*-mediated cross talk between Wnt and Shh signaling in CRC, which opens new molecular insights on existing interplay between these developmental pathways during carcinogenesis and therapy failure in CRC. Despite few studies having shown the role of *STIL* in various cancers, its association with cancer prognosis stands elusive. Our rectal tissue array analysis showed no association of *STIL* protein expression with invasive stages of tumor but was found to be highly expressed in early stages (**Figure 2** and **Table 2**). This again may be supported by our scratch assay experiment where we have observed that *STIL* silencing have no significant effect on HCT116 migration, and tumor xenograft also showed no tumor deposits in distant metastatic sites (**Supplementary Figure 2**). Further, overall survival (OS) and disease-free survival (DFS) analyses from c-Bioportal datasets revealed *STIL* overexpression to be significantly associated with lower DFS in CRC, but no association was found with OS (**Figure 1**). These observations show that *STIL* may not have a possible role in tumor invasion but could be critical in early tumorigenesis and therapy resistance, thus leading to lower DFS in CRC. However, more studies are warranted for elucidation of detailed molecular events mediated *via STIL* in CRC. Nonetheless, our study suggests Shh and Wnt regulation by *STIL* to be a possible mechanism governing its role in CRC (**Figure 6E**).

In conclusion, this report stands as the first study demonstrating an oncogenic role of *STIL* in CRC. Our study showed the role of *STIL* oncogene regulating CSC characteristics along with drug resistance properties, which had not been investigated to date in CRC. Further, *STIL* signs at a Shh-independent regulation of *β-catenin*, which we consider to be a novel finding, as regulation of Wnt by *STIL* opens a whole new prospective of research for Shh and Wnt cross talk in CRC. In addition, this study also sheds light on prognostic implications of *STIL* in CRC, which ensue to be very critical and imperative from a clinical perspective. Association of *STIL* with poor disease-free survival suggests its potency for a prognostic biomarker in CRC. Though the mechanistic proof of *STIL* function in details remains elusive, our study urges that *STIL* could be an important oncogene with an inherent ability to regulate multiple aspects of cancer progression in CRC, and it could be a warranted target for therapeutic intervention and prognostic marker in the future.

## Data Availability Statement

The raw data supporting the conclusions of this article will be made available by the authors, without undue reservation.

## Ethics Statement

All human biopsies were collected at Regional cancer center, Trivandrum, Kerala, India after approval of the Regional Cancer center Ethical committee (HEC No.43/2011) and from donors that signed written informed consent. The patients/participants provided their written informed consent to participate in this study. All the experiments on NOD-SCID mice were performed after approval from the Institute Animal Ethics Committee (IAEC/683/ASN/28).

## Author Contributions

TP conceived, planned, and carried out the experiments, and lead in data analysis and manuscript writing. VK and SJ assisted in planning and execution of xenograft experiment. ES and KR performed immunoblots and gave critical comments to improve work and also helped in article corrections. JV helped with statistical analysis of IHC tissue array data. CK performed the surgery and helped in obtaining the human biopsy samples and their storage. AS helped in RNA isolation from biopsies and qPCR. SN conceived, designed the project, and supervised its execution, manuscript proofreading, and provided critical feedbacks in manuscript writing. All authors contributed to the article and approved the submitted version.

## Funding

This work was supported by the Department of Biotechnology, Government of India (grant no-BT/PR3223/BRB/10/964/2011) and research fellowship to TP from Department of Biotechnology, Government of India.

## Conflict of Interest

The authors declare that the research was conducted in the absence of any commercial or financial relationships that could be construed as a potential conflict of interest.

## Publisher’s Note

All claims expressed in this article are solely those of the authors and do not necessarily represent those of their affiliated organizations, or those of the publisher, the editors and the reviewers. Any product that may be evaluated in this article, or claim that may be made by its manufacturer, is not guaranteed or endorsed by the publisher.
