# Supplementary material for: STIL Endows Oncogenic and Stem-Like Attributes to Colorectal Cancer Plausibly by Shh and Wnt Signaling
Source: Front Oncol. 2021 Aug 12;11:581671. doi: 10.3389/fonc.2021.581671 (PMC8416176; doi:10.3389/fonc.2021.581671)
Supplement: Supplementary file 1 [file DataSheet_1.docx]

**Supplementary Figure 1.**

**
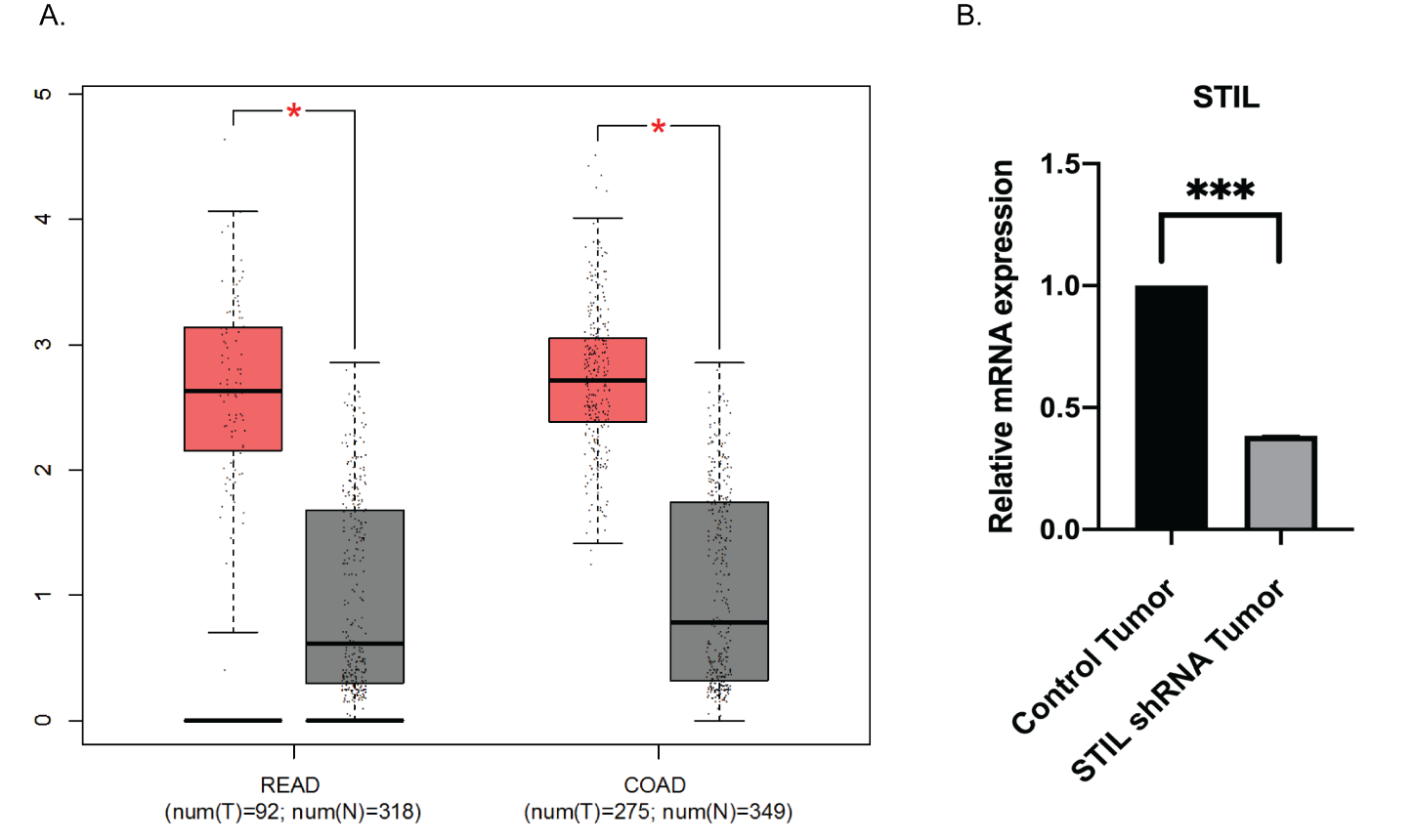
**

**Supplementary Figure 1.** (**A**) STIL m-RNA expression in normal colon and tumor obtained from TCGA datasets. (READ – Rectal Adenocarcinoma, COAD – Colon Adenocarcinoma). (**B**) Immunoblot showing expression of STIL in control and STIL-silenced xenograft tumors. Unpaired t-test results showing p value ≤0.001 are represented by ***.

**Supplementary Figure 2.**

**
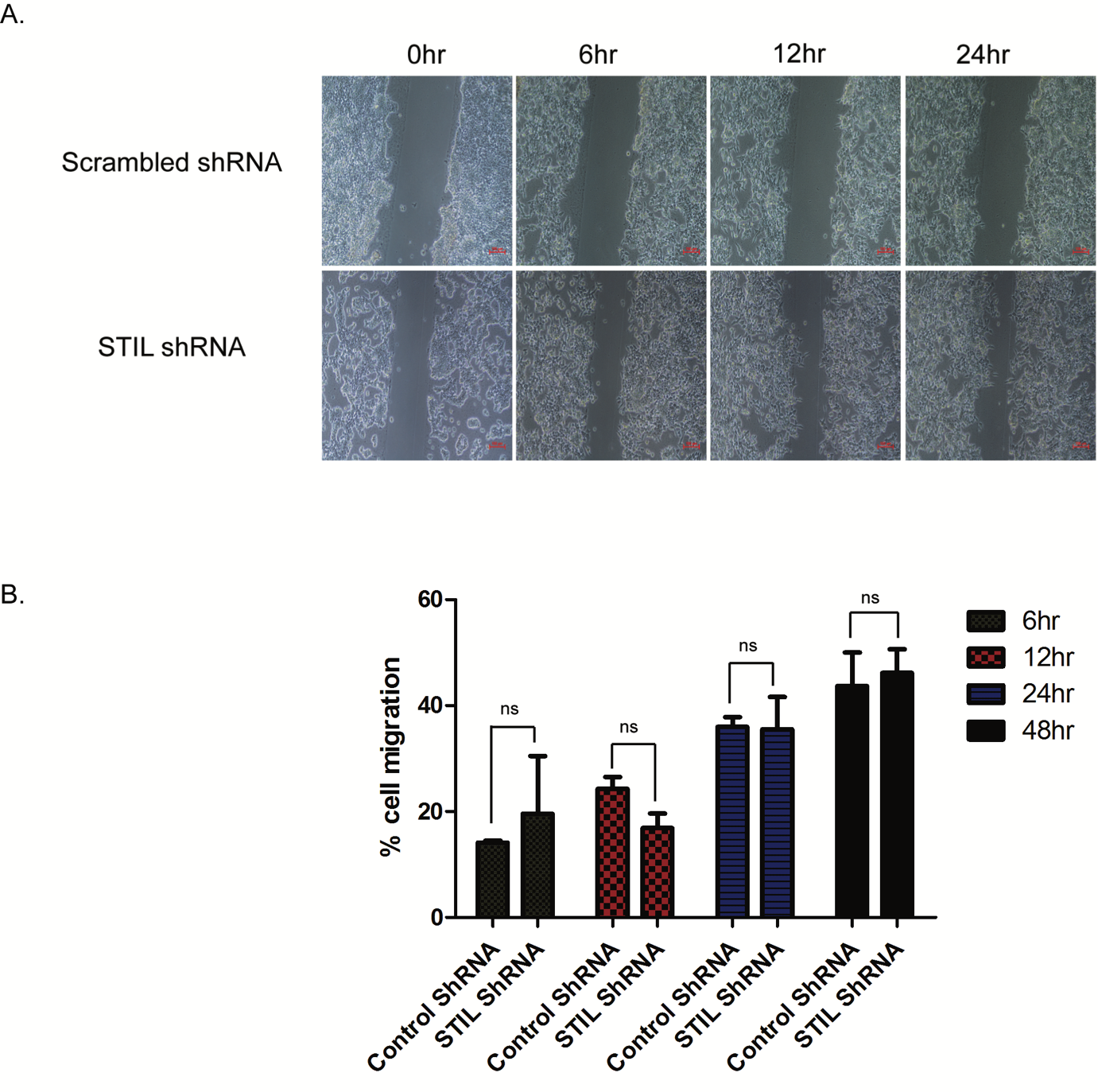
**

**Supplementary Figure 2. Effect of STIL silencing on cell migration**. (**A&B**) Cell migration in control and STIL silenced HCT116 cells at different time points.

**Supplementary Figure 3.**

**
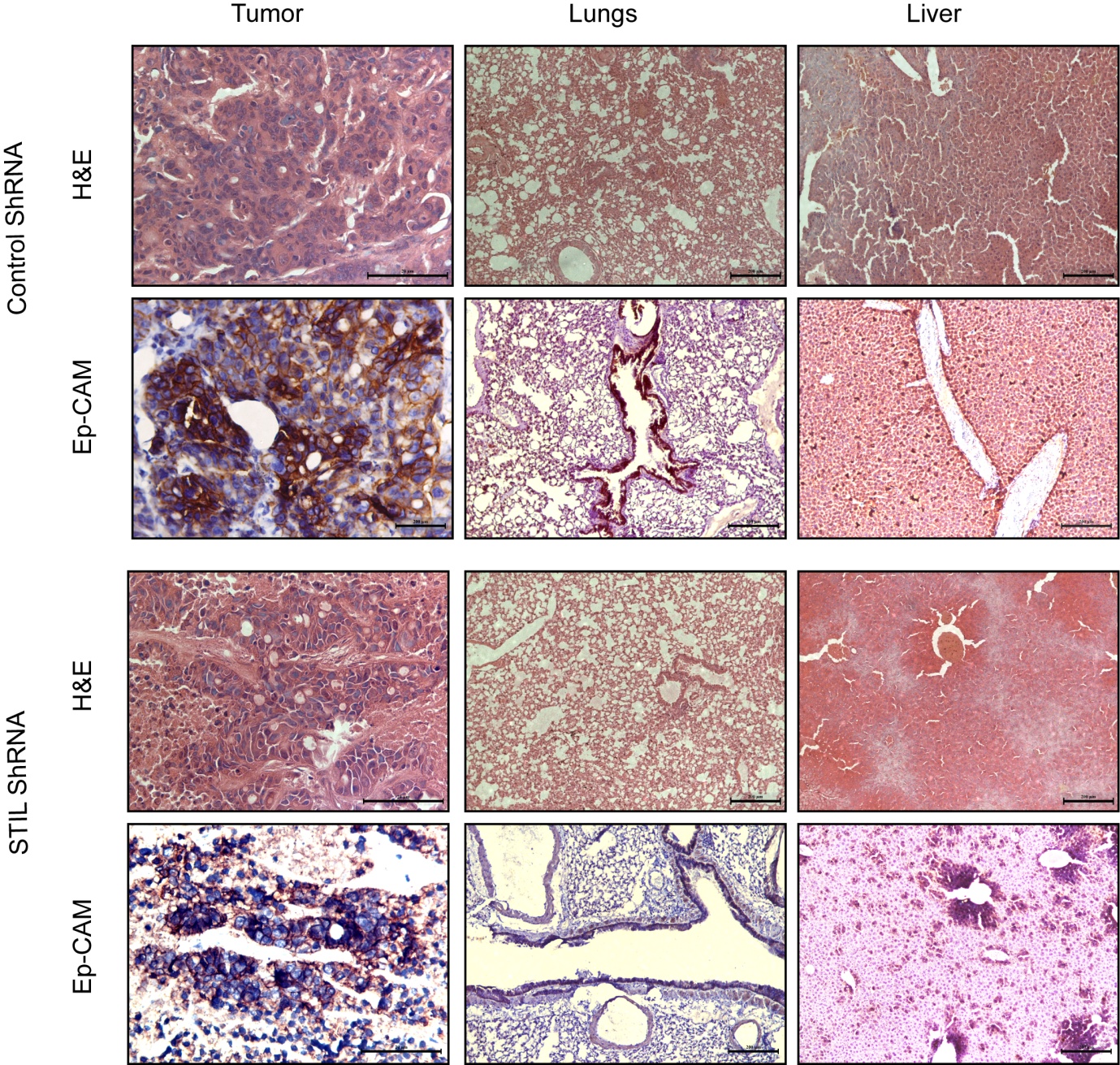
**

**Supplementary Figure 3.** Ep-CAM and H&E staining of control vs STIL silenced tumor xenograft, lungs and liver.

**Supplementary Figure 4.**

**
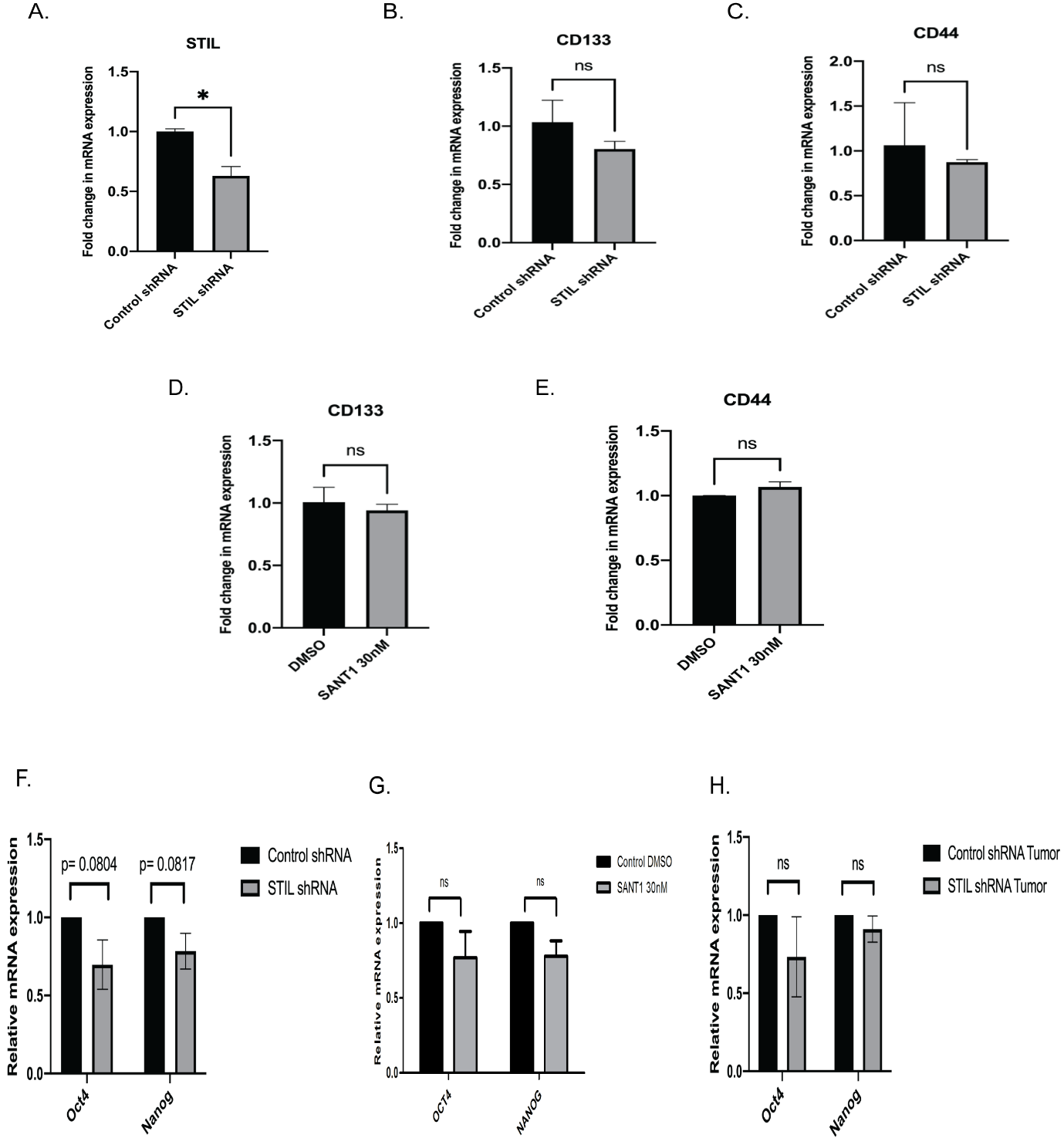
**

**Supplementary Figure 4. Effect of STIL silencing and Shh inhibition on stem cell markers.** Real time qPCR data showing expression of (**A-E**) CD133 & CD44 in CRC upon STIL silencing and SANT1 treatment in HCT116 cells. (**F&G**) OCT4 & NANOG in CRC upon STIL silencing and SANT1 treatment in HT-29 cells. (**H**) OCT4 & NANOG expression in STIL silenced H2-29 xenograft tumor. Unpaired t-test results showing p value ≤0.05, is represented by *, ns – not significant.

**Supplementary Figure 5.**


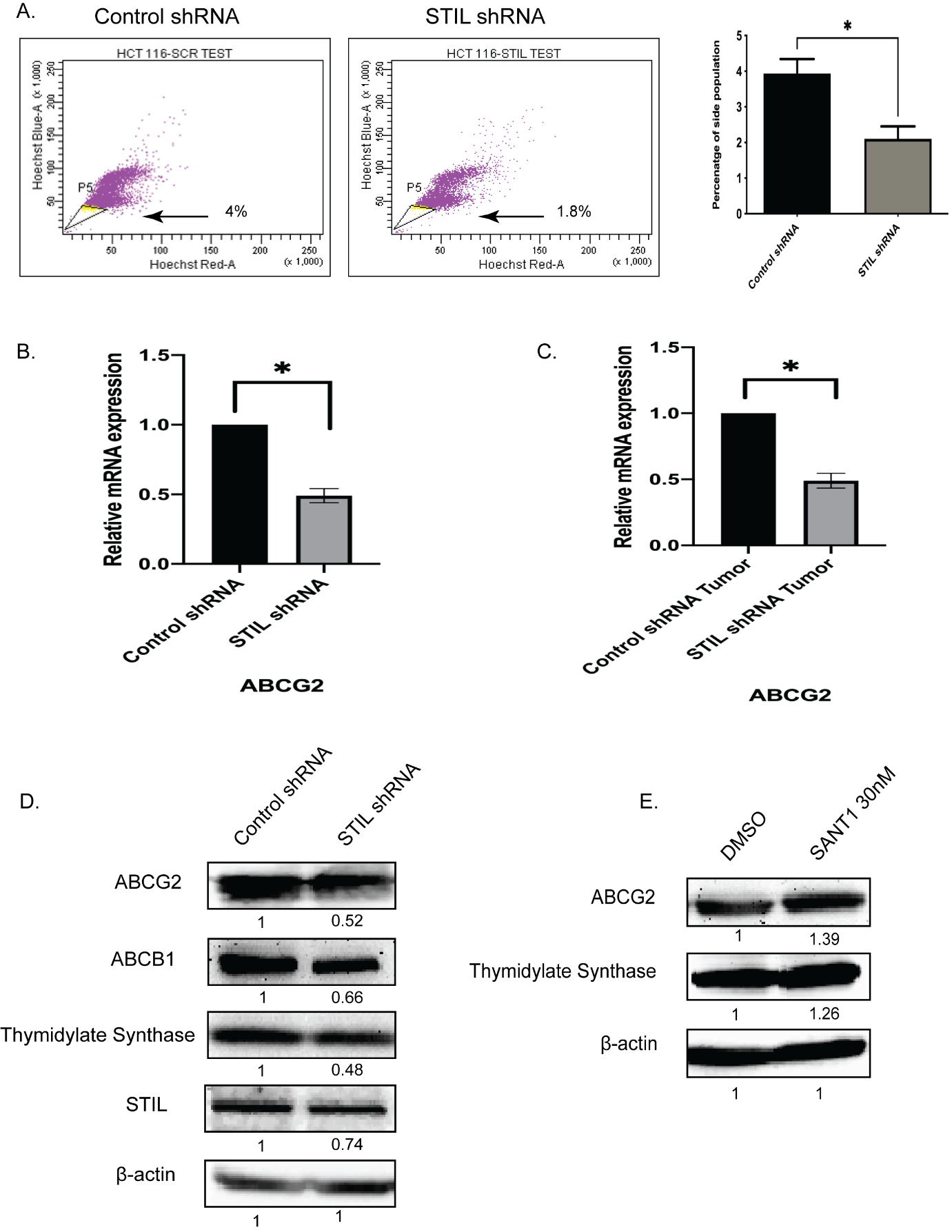


**Supplementary Figure 5. Effect of STIL silencing on drug efflux in CRC.** (**A**) Dot plot showing side population cells in control and STIL silenced HCT116 cells and bar graph representing quantification of the same. (**B**) ABCG2 expression in control and STIL silenced HT29 cells. (**C**) ABCG2 expression in control and STIL silenced Xenograft tumors (**D**) Expression of drug efflux proteins and Thymidylate synthase in control and STIL silenced HCT116 cells. (**E**) Expression of ABCG2 and Thymidylate synthase in control and SANT1 treated HCT116 cells. Unpaired t-test results showing p value ≤0.05 is represented by *.

**Supplementary Figure 6.**

**
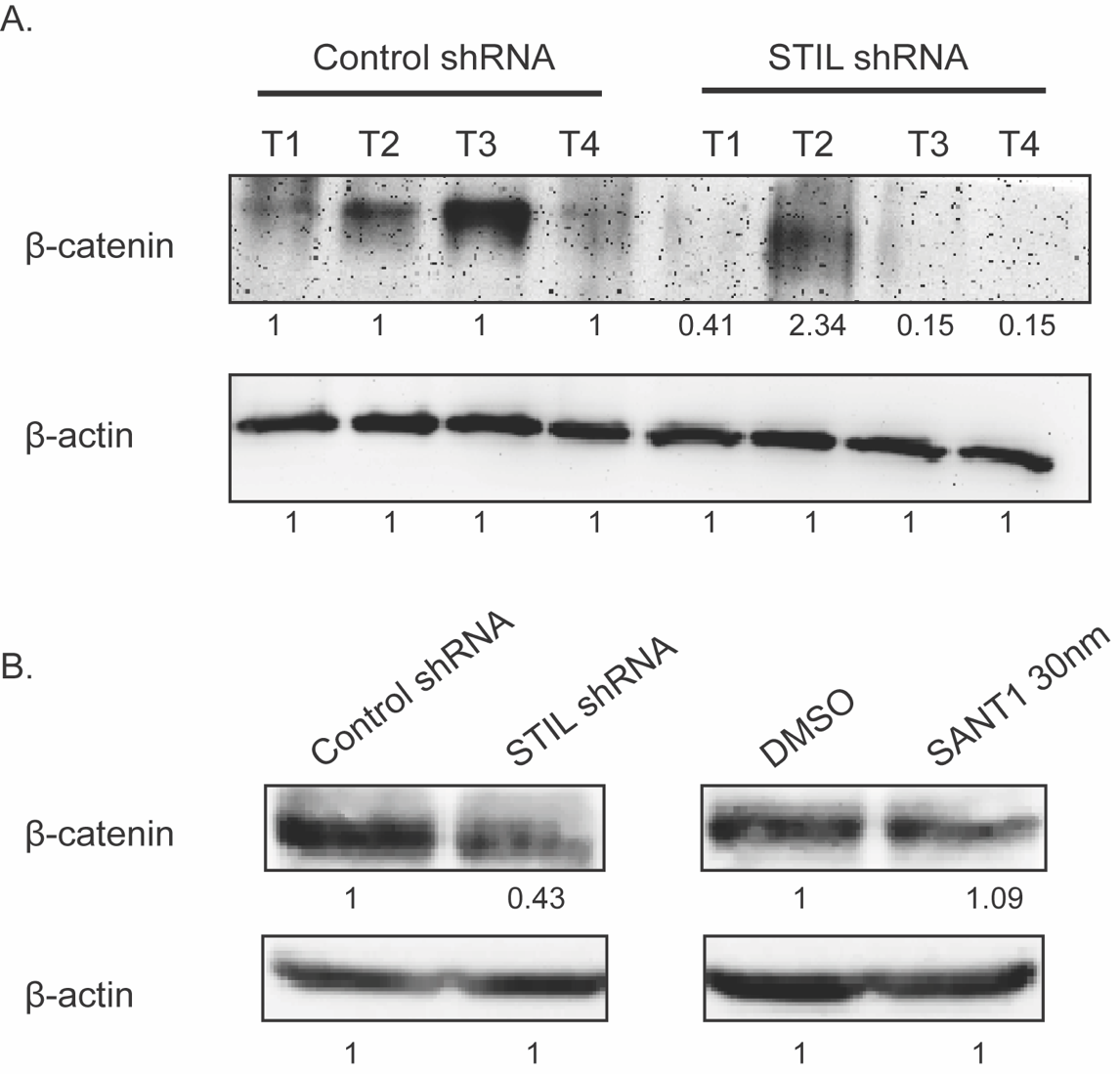
**

**Supplementary Figures 6. Showing expression of β-catenin protein upon STIL silencing.** (**A**) Expression of β-catenin in control and STIL silenced tumor xenograft. (T- Tumor, 1-4 represents tumors from different mice). (**B**) Expression of β-catenin in STIL silenced and SANT1 treated HCT116 cells.

**Supplementary Figure 7.**

**
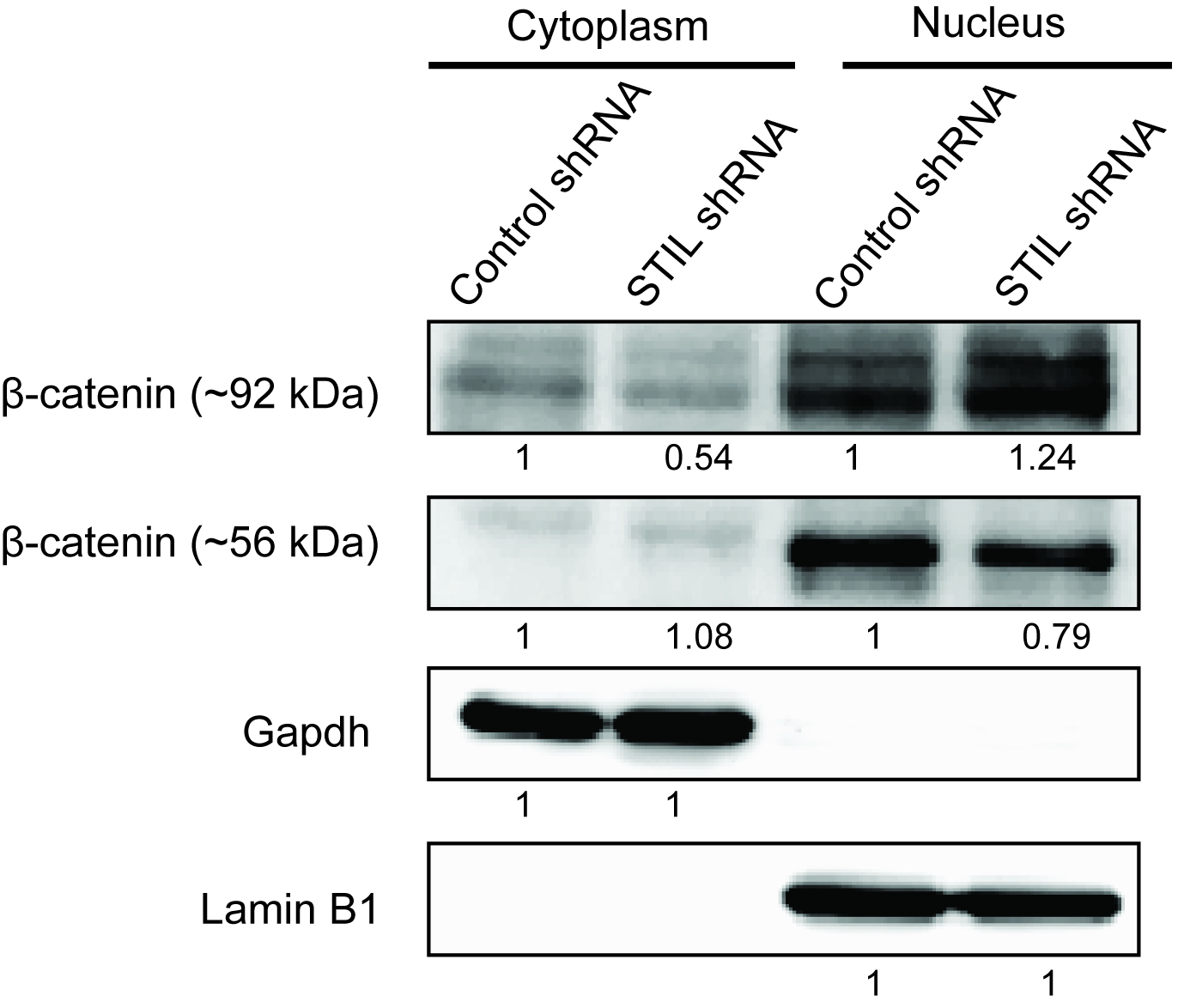
**

**Supplementary Figure 7. Showing expression of β-catenin protein in cytoplasmic and nuclear fraction upon STIL silencing.** Western blot showing β-catenin protein levels in cytoplasm and nucleus in control shRNA and STIL shRNA treated HT29 cells (The 92 kDa band corresponds to full-size Beta-catenin and 56 kDa band corresponds to cleaved active fragment of Beta-catenin).
